# Acoustic Focusing Enhancement In Fresnel Zone Plate Lenses

**DOI:** 10.1038/s41598-019-43495-x

**Published:** 2019-05-08

**Authors:** Daniel Tarrazó-Serrano, Sergio Pérez-López, Pilar Candelas, Antonio Uris, Constanza Rubio

**Affiliations:** Centro de Tecnologías Físicas, Universitat Politècnica de València, Camí de Vera s/n, 46022, València, Spain

**Keywords:** Engineering, Acoustics

## Abstract

The development of flat acoustic lenses for different applications such as biomedical engineering is a topic of great interest. Flat lenses like Fresnel Zone Plates (FZPs) are capable of focusing energy beams without the need of concave or convex geometries, which are more difficult to manufacture. One of the possible applications of these type of lenses is tumor ablation through High Intensity Focused Ultrasound (HIFU) therapies with real time Magnetic Resonance Imaging (MRI) monitoring. In order to be MRI compatible, the FZP material cannot have electromagnetic interaction. In this work, a Phase-Reversal FZP (PR-FZP) made of Polylactic Acid (PLA) manufactured with a commercial 3D printer is proposed as a better, more efficient and MRI compatible alternative to conventional Soret FZPs. Phase-Reversal lenses, unlike traditional FZPs, take advantage of all the incident energy by adding phase compensation regions instead of pressure blocking regions. The manufactured PR-FZP achieves 21.9 dB of focal gain, which increases the gain compared to a Soret FZP of its same size by a factor of 4.0 dB. Both numerical and experimental results are presented, demonstrating the improved focusing capabilities of these types of lenses.

## Introduction

One of the most important issues in the acoustics field is sound focusing due to its multiple applications. This focalization phenomenon is produced by acoustic lenses, which are devices capable of concentrating acoustic energy in a specific area. Acoustic lenses are used in many applications of different nature, ranging from medical applications for the diagnosis and/or treatment of medical pathologies, to non-destructive testing or food industry^1,2^. This wide range of applications of acoustic lenses makes these devices a hot topic among the scientific community. Sound focusing can be achieved through diffraction or refraction phenomena. Thus, in the last decades, different designs of acoustic lenses have been devised, such as acoustic lenses based on phononic crystals^3^, gradient acoustic lenses that use space-coiling in their designs^4,5^, acoustic lenses that use Helmholtz resonators or metamaterial based split-ring type resonators in their designs^6^ or acoustic lenses based on Fabry-Perot resonances in apertures^7^. Now, the geometry and efficiency in the optimal design of an acoustic lens are crucial parameters for its subsequent application to different fields. An optimal design would be one in which the lens had a small size, flat geometry and great energy efficiency. In this sense, recent studies have developed small size and high transmission efficiency flat ultrasound lenses based on sandwiched layers of silicone and resin^8^.

Acoustic lenses focus sound in the same way than optical lenses focus light, because the underlying theory is applicable to both mechanical and electromagnetic waves. The research and the devices obtained for the case of electromagnetic waves can be extended to acoustic waves. One of these devices, that originally was conceived to focus electromagnetic waves and that was later transferred to the acoustics field, is the Fresnel Zone Plate (FZP) lens. FZP lenses are formed by a set of concentric rings with decreasing width. Each ring constitutes a Fresnel region, and between two consecutive regions there is a *π* phase difference. Based on this fact, two types of lenses can be distinguished: Soret type FZPs and Phase-Reversal FZPs (PR-FZPs). Soret type FZPs^9^ alternate transparent with pressure blocking regions that reflect the pressure contributions that are in phase opposition with those of the transparent regions. Blocking regions are implemented with materials that have either a high impedance contrast with the host medium or a high attenuation constant, which ensures a high reflection and a low transmission coefficient, respectively. PR-FZPs^10^ replace blocking regions with phase reversal regions that correct the phase of the pressure contributions by adding a *π* phase change that generates a constructive interference at the focal distance. This means that, unlike FZPs, with PR-FZPs all the regions of the lens and not only the transparent ones contribute constructively to the focal area, which ideally increases the lens efficiency and focal intensity by a factor of 4, as twice the pressure is focused into the focal distance.

In general, FZP lenses can be used for acoustic focusing in both air and water. In this sense, Shindel^11^ and Welter^12,13^ designed and presented experimental results on FZP lenses for their use in air. Subsequently, the concept of coiling up space, this is labyrinthine channels, was applied to the focusing of sound waves in air. Moleron *et al*.^14^ and Li *et al*.^15^ increased the efficiency of FZP lenses using labyrinthine channels in the opaque rings. In the case of underwater acoustic focusing, Calvo *et al*.^16^ proposed a FZP lens with alternating transparent and opaque zones made of soft silicone rubber. Recently, studies about the influence of the reference radius or phase in the FZPs design have been carried out. Using this parameter in the implementation of FZP lenses, their focus properties can be modified^17^. Moreover, side-lobe levels can be improved to obtain a better spatial resolution^18^. Generally, when FZP lenses are used in air or water, these are manufactured using materials with a large impedance mismatch with the host medium, which is the main reason why metals are generally used for their implementation. One of the main uses of acoustic lenses in the medical field is tumor ablation through High Intensity Focused Ultrasound (HIFU) therapies. These therapies are usually guided in real time using MRI monitoring systems, which can provide an image of the temperature rise in the targeted cancerous tissue^19^. However, MRI systems are not compatible with metallic devices, which makes FZP implementations more difficult to achieve. Many of the compatible MRI commercial devices use PolyLactic Acid (PLA) as material^20^. PLA is an Aliphatic Polyester derived from 100% renewable resources, versatile and biodegradable with reduced costs, and it is used in biomedical applications^21^.

Commercial HIFU devices are either based on phased arrays or geometrically focused transducers^22,23^. On the one hand, phased arrays offer high flexibility with dynamic control of the focal distance and beam width, although these devices are usually very expensive. On the other hand, focused transducers have lower cost compared to phased array solutions, but there is a lack of control in the focal distance, which can not be practical in some therapeutic situations. In this sense, FZPs are simpler options that allow to control both the focal distance and the beam width. The focal distance of FZPs can be dynamically shifted by tuning the working frequency^24^, while the number of Fresnel regions selected at the design stage of the lens controls the beam width.

Nowadays, acoustic FZPs are based on Soret type implementations, which focusing efficiency is low because half of the incident energy is reflected at the blocking regions. In this paper, we design, manufacture and experimentally validate a PR-FZP lens, which design is inspired in a phase-reversal zone plate^25^. The PR-FZP, originally developed for electromagnetic waves, has been used as an antenna in the range of microwave and millimeter wave regions^26,27^, and more recently as THz sieves for optical focusing applications^28^. Knowing that we can extend the results obtained for electromagnetic waves to acoustic waves, an acoustic PR-FZP has been designed to increase the energy efficiency compared to conventional FZPs. In addition, it is made of a MRI compatible material, which makes the PR-FZP suitable for therapeutic ultrasound focusing applications. The acoustic PR-FZP has been manufactured using a commercial 3D printer and PLA. Both numerical and experimental results indicate excellent performance, obtaining gains at the focal region above 21.9 dB. Furthermore, due to it its low manufacturing cost and low weight, the designed PR-FZP offers great flexibility, becoming an appealing alternative to conventional HIFU devices.

## Results

When a piston transducer is used as emitter, spherical wave incidence on the lens has to be considered. In the far field, the piston can be described as a point source emitter with a given directivity pattern, *D*(*θ*)^29^. The design equation of the FZP radii can be obtained considering a *λ*/2 increase between the pressure propagation paths of two consecutive regions, which is equivalent to a *π* phase increase. Thus, the radii can be calculated using the following expression^16,30^:

1 $$d+F+\frac{n\lambda }{2}=\sqrt{{r}_{n}^{2}+{d}^{2}}+\sqrt{{r}_{n}^{2}+{F}^{2}},$$

where *d* is the distance between the point source and the FZP, *F* is the focal distance, *λ* is the wavelength, *r*_*n*_ is the radius of each region and *n* = 1, 2, 3, ..., *N*, being *N* the total number of Fresnel regions.

Once the different radii are calculated using Eq. 1, the FZP is obtained by alternating transparent regions with either pressure blocking (Soret FZP) or phase-reversal regions (PR-FZP). If a PR-FZP implementation is selected, the thickness of the phase-reversal regions has to be such that the phase difference introduced compared to transparent regions is an odd multiple of *π*. The phase difference is given by Δ*θ* = |*k*_*m*_ − *k*_0_|*t*_*h*_, where *t*_*h*_ is the thickness of the lens and *k*_*m*_ = 2*π*/*λ*_*m*_ and *k*_0_ = 2*π*/*λ*_0_ are the wave numbers of the lens material and the host medium, respectively. Therefore, the thickness of the lens can be calculated as

2 $${t}_{h}=\frac{q}{|{k}_{m}-{k}_{0}|}\pi =\frac{q}{2}\frac{{\lambda }_{0}{\lambda }_{m}}{|{\lambda }_{0}-{\lambda }_{m}|},$$

where *q* = 1, 3, 5, ... is a design parameter that determines the thickness of all Fresnel regions.

**Figure 1:**
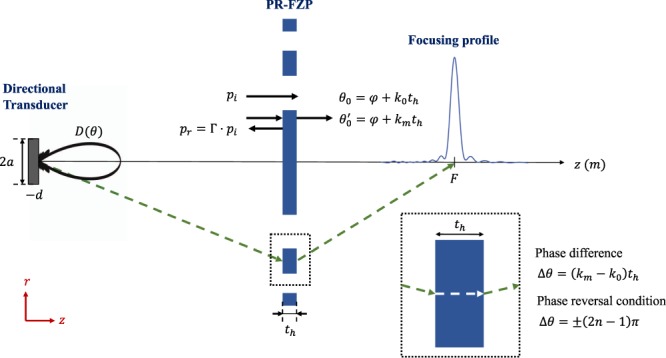
Schematic diagram of the Phase-Reversal Fresnel Zone Plate (PR-FZP).

Figure 1 shows a scheme of a PR-FZP placed at a distance *d* from a directional piston transducer. As it can be observed, the lens material introduces a phase shift to the incident pressure, which generates a constructive interference at the focal distance. Ideally, the pressure generated at the focal distance by a PR-FZP is twice the pressure generated by a conventional FZP because every Fresnel region of the lens contributes constructively at the focus. However, as depicted in Fig. 1, in a practical case some of the incident energy is reflected at the lens material due to the impedance mismatch between the material and the host medium, which means that the pressure at the focal distance, and therefore the lens efficiency, will be reduced compared to the ideal case. The impedance mismatch can be evaluated using the reflection coefficient, defined as Γ = *p*_*r*_/*p*_*i*_, with *p*_*r*_ and *p*_*i*_ being the reflected and the incident pressure, respectively. In the situation described in Fig. 1, the reflection coefficient can be calculated using the following expression:

3 $${\rm{\Gamma }}=\frac{j\,\tan ({k}_{m}{t}_{h})({Z}_{m}^{2}-{Z}_{0}^{2})}{2{Z}_{m}{Z}_{0}+j\,\tan ({k}_{m}{t}_{h})({Z}_{m}^{2}+{Z}_{0}^{2})},$$

where *Z*_*m*_ = *ρ*_*m*_*c*_*m*_ and *Z*_0_ = *ρ*_0_*c*_0_ are the characteristic acoustic impedances of the lens material and the host medium, respectively. *ρ*_0_, *ρ*_*m*_, *c*_0_ and *c*_*m*_ are the densities and sound propagation speeds of the lens material and the host medium.

**Figure 2:**
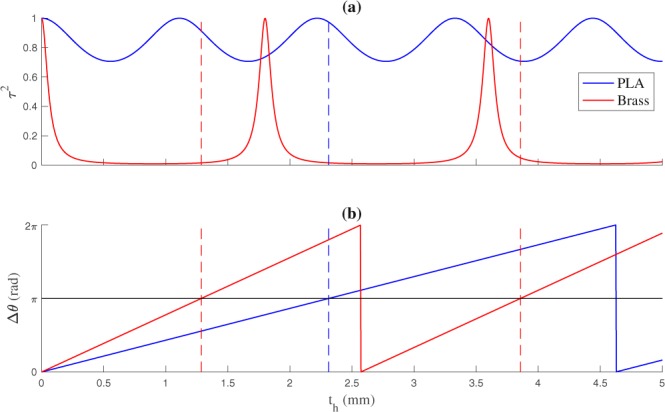
(**a**) Energy transmission coefficient as a function of the lens thickness and (**b**) phase difference between the host medium and the material. Blue lines correspond to PLA and red lines correspond to brass.

The transmission coefficient, defined as *τ* = |*p*_*t*_|/|*p*_*i*_|, where *p*_*t*_ is the transmitted pressure, can be calculated from the reflection coefficient as $\tau =\sqrt{1-|{\rm{\Gamma }}{|}^{2}}$. A low transmission coefficient would reduce the lens focusing capability. Thus, there are two design factors regarding the implementation of the phase-reversal regions: the lens material and its thickness. The lens material has to be selected in order to minimize the reflection coefficient, which maximizes the transmitted pressure through the phase-reversal regions. Once the material is selected, its thickness can be directly calculated using Eq. 2, which provides the phase-reversal condition. In this sense, Fig. 2(a) shows a comparison between the squared transmission coefficient of a lens made of PLA (blue line) and a lens made of brass (red line) as a function of the material thickness. The host medium is water, and the working frequency is fixed to 1 MHz. Table 1 shows the acoustic properties of the considered materials. Figure 2(b) depicts the phase difference between transparent and phase-reversal regions for both materials. The horizontal black line represents the Δ*θ* = *π* condition, while vertical dashed lines represent the thicknesses which fulfill the phase-reversal behaviour. As it can be observed, for the brass material, the *π* phase shift occurs at the thickness of 1.286 and 3.854 mm, when the energy transmission coefficient is 0.016 and 0.051, respectively. This means that, although the phase-reversal condition is met, more than 94% of the incident energy will be reflected due to impedance mismatch between water and brass. This is the main reason why brass is usually employed to implement the pressure blocking regions of Soret FZPs. In contrast, if the lens is implemented in PLA, the *π* phase shift occurs at a thickness of 2.313 mm, when the squared transmission coefficient is higher than 97%. This fact makes PLA a suitable material for implementing PR-FZPs in underwater focusing applications, as its acoustic impedance is similar to the water impedance.

**Table 1:** Acoustic properties of water, PLA and brass.

	*ρ* (kg/m^3^)	*c* (m/s)	*Z* (MRayl)	|Γ|
Water	1000	1500	1.5	N/A
PLA	1240	2220	2.753	0.17
Brass	8400	3600	30.24	0.99

The reflection coefficient is calculated for a PLA and brass thickness of 2.313 and 1.286 mm, respectively.

In order to demonstrate the improved acoustic focusing performance of 3D printed phase reversal lenses over conventional brass lenses, numerical simulations and experimental measurements have been carried out. Numerical simulations have been computed using COMSOL Multiphysics, which uses the Finite Element Method (FEM) to solve the wave equation in the different media. Experimental measurements have been developed using an automated 3D underwater positioning system. A needle hydrophone fixed to a programmable robotic arm is employed as receiver, while a directional piston transducer connected to a Pulser is employed as transmitter. The separation distance between lens and transducer is *d* = 340 mm, which is not enough to consider plane wave incidence and therefore the spherical wave design approach must be used. Additional details of the FEM model and the experimental set-up can be found at the Methods section.

**Figure 3:**
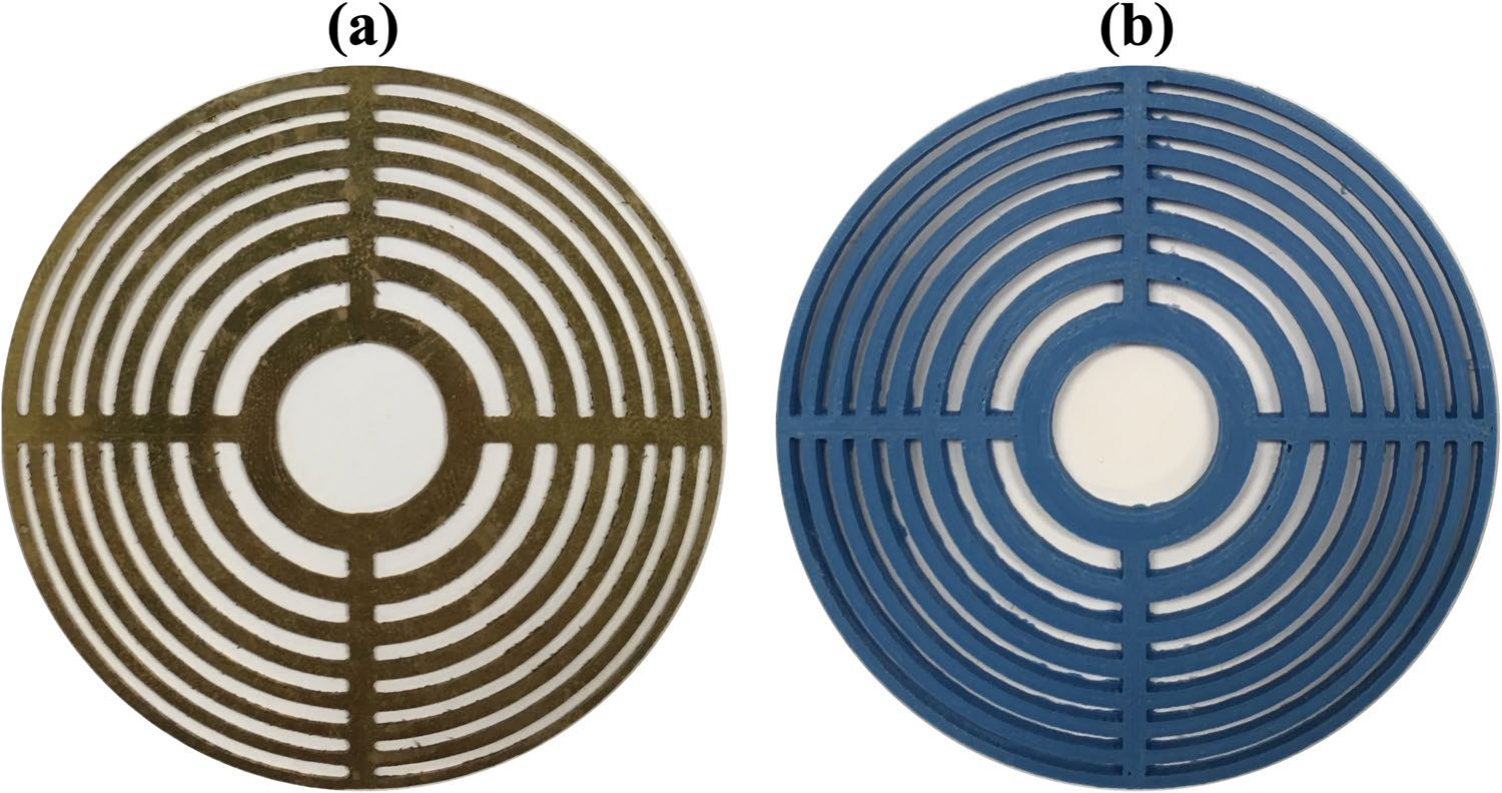
Manufactured lenses: (**a**) FZP made of brass and (**b**) PR-FZP made of PLA.

Two different lenses have been manufactured, being one of them a conventional Soret FZP made of brass and the other a 3D printed PR-FZP made of PLA. Figure 3 shows both lenses, where Fig. 3(a) corresponds to the FZP and Fig. 3(b) corresponds to the PR-FZP. The PLA thickness is *t*_*h*_ = 2.313 mm, which provides the phase-reversal behaviour, as mentioned above. The brass thickness is *t*_*h*_ = 0.5 mm, which ensures a high reflection coefficient and therefore a good pressure blocking behaviour. For both lenses, the working frequency is 1 MHz, the focal distance is *F* = 50 mm and the number of Fresnel regions is *N* = 16, considering a source separation distance of *d* = 340 mm. As it can be observed from Fig. 3, both lenses have been manufactured with a central transparent zone instead of a central blocking/phase-reversal region. This design choice is selected as a consequence of the piston transducer, because with a central blocking region the main energy contribution of the transducer would be reflected, and with a phase-reversal region the energy transmission coefficient would be reduced.

**Figure 4:**
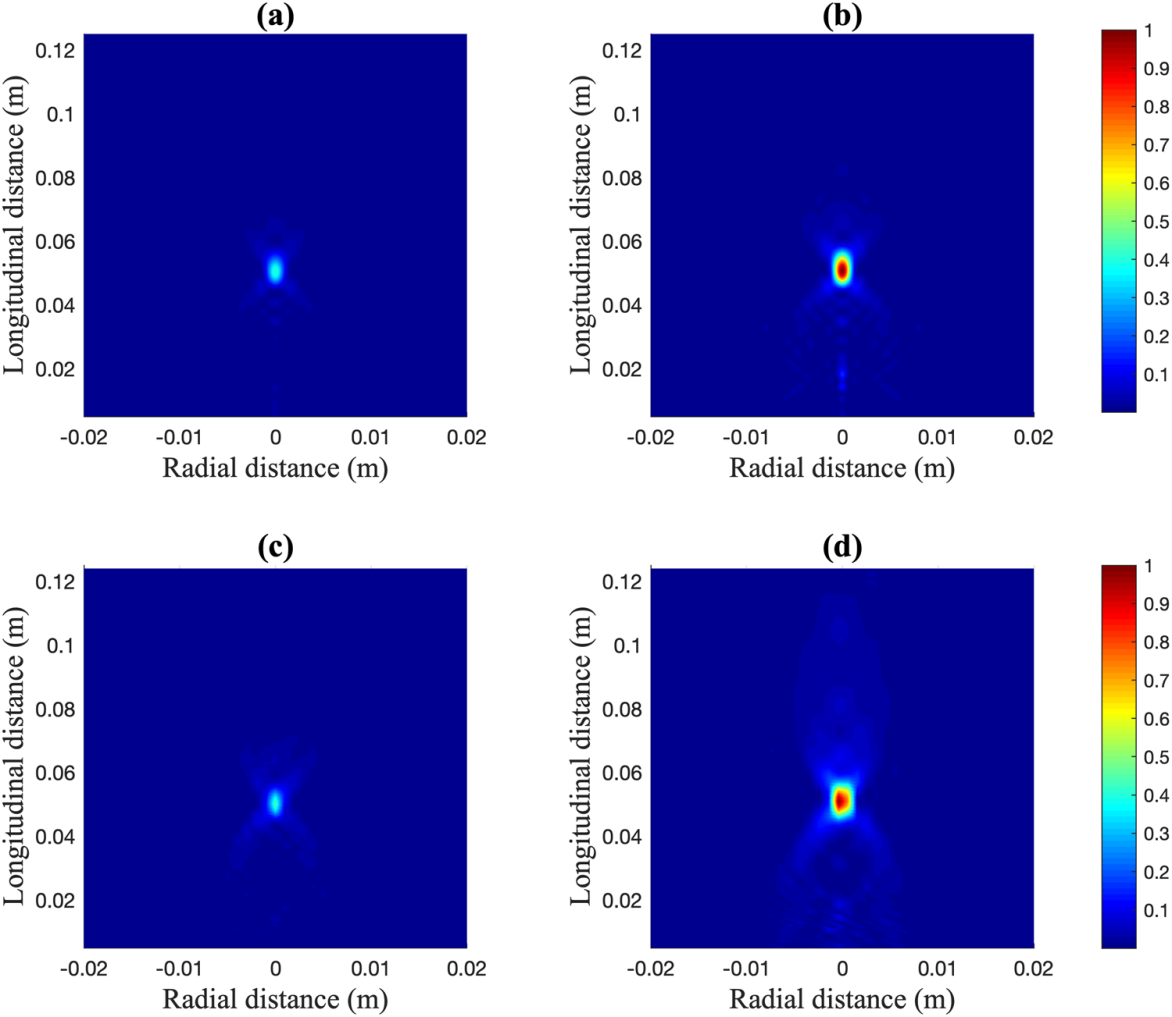
Measured intensity maps: FZP simulated (**a**) and measured (**c**), PR-FZP simulated (**b**) and measured (**d**).

Figure 4 shows acoustic intensity maps normalized to the global maximum, which is achieved in the PR-FZP case. Figure 4(a,b) correspond to simulation results, while Fig. 4(c,d) correspond to experimental measurements. As it can be observed, numerical and experimental results agree very well, and the PLA PR-FZP lens (Fig. 4(b,d)) achieves a higher focal intensity compared to the conventional brass FZP (Fig. 4(a,c)). The differences found between both lenses, in addition to those already mentioned above, are found in the size of the focus and the energy surrounding it. In the case of the conventional FZP, almost all the energy is concentrated in the focal area for both experimental and numerical results, while in the case of the PR-FZP the experimental measurements show that the focal area is increased compared with the numerical results. This fact is due to the effect of the cross-shape structural support of the PR-FZP, which is not included in the simulation model. This support introduces a *π* phase change at transparent Fresnel regions that generates a destructive interference at the focal distance, which increases the size of the focus in both longitudinal and radial directions, and therefore decreases the focusing efficiency of the lens. For the conventional FZP, the cross-shape structural support does not interfere destructively at the focal distance as the pressure is reflected at the brass, and therefore the focal area is not increased as in the PLA case.

**Figure 5:**
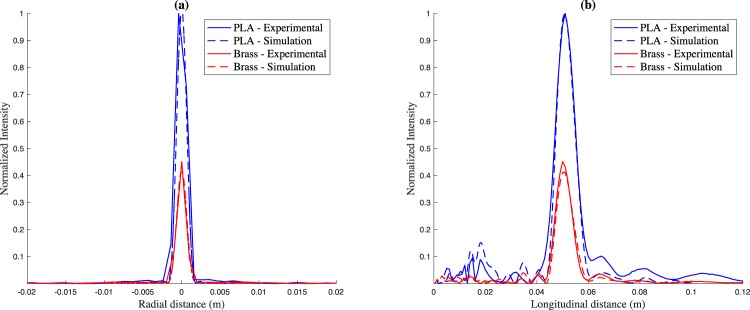
(**a**) Normalized radial intensity and (**b**) normalized longitudinal intensity for PR-FZP made of PLA (blue) and FZP made of brass (red). Solid lines correspond to experimental results, while dashed lines correspond to simulation results.

Figure 5(a,b) depict radial and longitudinal intensity cuts for both numerical and experimental results, respectively. Once again, the results are normalized to the maximum intensity value. An additional parameter to evaluate the focusing improvement factor of PR-FZPs over conventional FZPs can be defined as the ratio between the focal intensity of the PR-FZP and the focal intensity of the conventional FZP. Thus, as it can be seen in Fig. 5(a,b), a considerable improvement factor of 220% is achieved when using the Phase-Reversal lens. Figure 5(a) also depicts an almost absence of secondary lobes for both lenses. This fact is due to the influence of the piston directional transducer, as its directivity pattern has an effect similar to the FZP apodization effect achieved through efficiency decreasing regions^31^, which reduces the secondary lobes. Focusing profiles of Fig. 5(b) show that both lenses focus at the designed *F* = 50 mm focal distance, which means that the spherical wave incidence design approach works when using piston transducers.

One interesting parameter that can be used to characterize the focusing performance of a lens is the focusing efficiency. In this case, the focusing efficiency, *η*_*F*_, is defined as the ratio between the energy at the focal area and the incident energy at the lens. The focal area is given by the focal radius, which is the radial distance which contains the main focusing lobe. Additional explanation of focusing efficiency calculations can be found in the Methods section. If an ideal model is considered, theoretical maximum focusing efficiencies of 12.2% and 42.1% are obtained for the conventional FZP and the PR-FZP, respectively. These values are indicators that, ideally, a PR-FZP could reach approximately four times the energy efficiency of a conventional FZP.

Table 2 shows a comparison between numerical and experimental results of the main focusing parameters of both lenses. The focal gain has been calculated as the ratio between the intensity achieved with the lens and the intensity measured in absence of the lens. As it can be observed, the manufactured PR-FZP achieves a focal gain of 21.9 dB, which results in an increase of 4.0 dB over the conventional FZP. The measured FLHM is 5.174*λ* for the FZP case and 5.87*λ* for the PR-FZP case, while the FWHM measured values are 0.87*λ* and 1.27*λ* for the FZP and PR-FZP case, respectively. This means that, as explained above, the focal area is increased in terms of both FLHM and FWHM when the PLA lens is employed instead of the brass lens. On the other hand, the focusing efficiency achieved with the PR-FZP is 30.20%, which results in more than twice the focusing efficiency achieved with the FZP (11.33%). If numerical and experimental results are compared, good agreement between FEM and measured parameters is observed. However, a slight difference between the FWHM value obtained from the FEM model and the experimental measurement can be found for the conventional FZP case. This phenomenon could be caused by the limitation in resolution of the experimental set-up. As explained in the Methods section, the experimental set-up has a spatial resolution of 1 × 1 × 1 mm^3^. At the working frequency of the lens (1 MHz), the host wavelength is *λ* = 1.5 mm, which is very close to the spatial resolution of the measurement system and therefore limits the accuracy of the measured FWHM values, which are approximately *FWHM* ≅ *λ*.

**Table 2:** Simulated (FEM) and measured (Exp.) focusing characteristics of the FZP and PR-FZP.

	Gain (dB)	FLHM (*λ*)	FWHM (*λ*)	*η*_*F*_ (%)
FZP (FEM)	18.59	5.155	1.02	12.10
FZP (Exp.)	17.90	5.174	0.87	11.33
PR-FZP (FEM)	22.44	5.480	1.01	32.40
PR-FZP (Exp.)	21.90	5.870	1.27	30.20

## Discussion

In this work, the design process and focusing properties of acoustic PR-FZP lenses have been analyzed. Simulation and experimental results are presented, and they show that 3D printed PR-FZPs made of PLA can achieve higher focusing efficiency and focal intensity gain than conventional Soret FZPs, with an improvement in both factors over 200%. The simulation results are experimentally validated, showing a good agreement between the FEM model and the measured intensity maps.

PR-FZPs implemented in PLA grant affordable acoustic lenses with high focusing efficiency. The 3D printing technology allows a reduction of the manufacturing costs compared to metal implemented lenses. In the medical field, acoustic lenses manufactured using 3D printing techniques result very useful due to their flexibility, since this type of lenses can be designed and built specifically for each treatment in a few hours. Moreover, these lenses could also be used in MRI environments due to its manufacturing material. PLA is a biocompatible material without electromagnetic interaction that has already been used in medical environments due to its structural qualities. Therefore, these PR-FZP lenses could be used in ultrasound focusing therapies with MRI real time monitoring with the characteristic of being able to be easily designed and manufactured *in situ*.

## Methods

### Focusing efficiency

Diffraction is a phenomenon that occurs when an acoustic wavefront impinges on an obstacle with an aperture or border. The acoustic intensity generated by a lens when the field is diffracted at its apertures can be calculated using the non-paraxial diffraction integral, which is given by

4 $$I(z,r)=\frac{1}{{\lambda }^{2}}{|{\int }_{0}^{2\pi }{\int }_{0}^{{r}_{N}}q(r^{\prime} ){p}_{i}(r^{\prime} )\frac{r^{\prime} }{R}{e}^{-jkR}dr^{\prime} d\phi |}^{2},$$

where *p*_*i*_(*r*′) is the incident pressure at the lens, *q*(*r*′) is the pupil function of the lens, *r*′ is the radial axis at the surface of the lens, *r*_*N*_ is the external radius of the lens, and $R=\sqrt{{r}^{2}+{(r^{\prime} )}^{2}+{z}^{2}-2rr^{\prime} \,\cos \,\phi }$, being *φ* the rotation angle at the surface of the lens. The pupil function describes the geometry of the lens. For an ideal Soret lens, the pupil function is 1 at transparent regions and 0 at pressure blocking regions, while for an ideal phase reversal lens the pupil function at phase reversal region is −1, which models the *π* phase shift.

**Figure 6:**
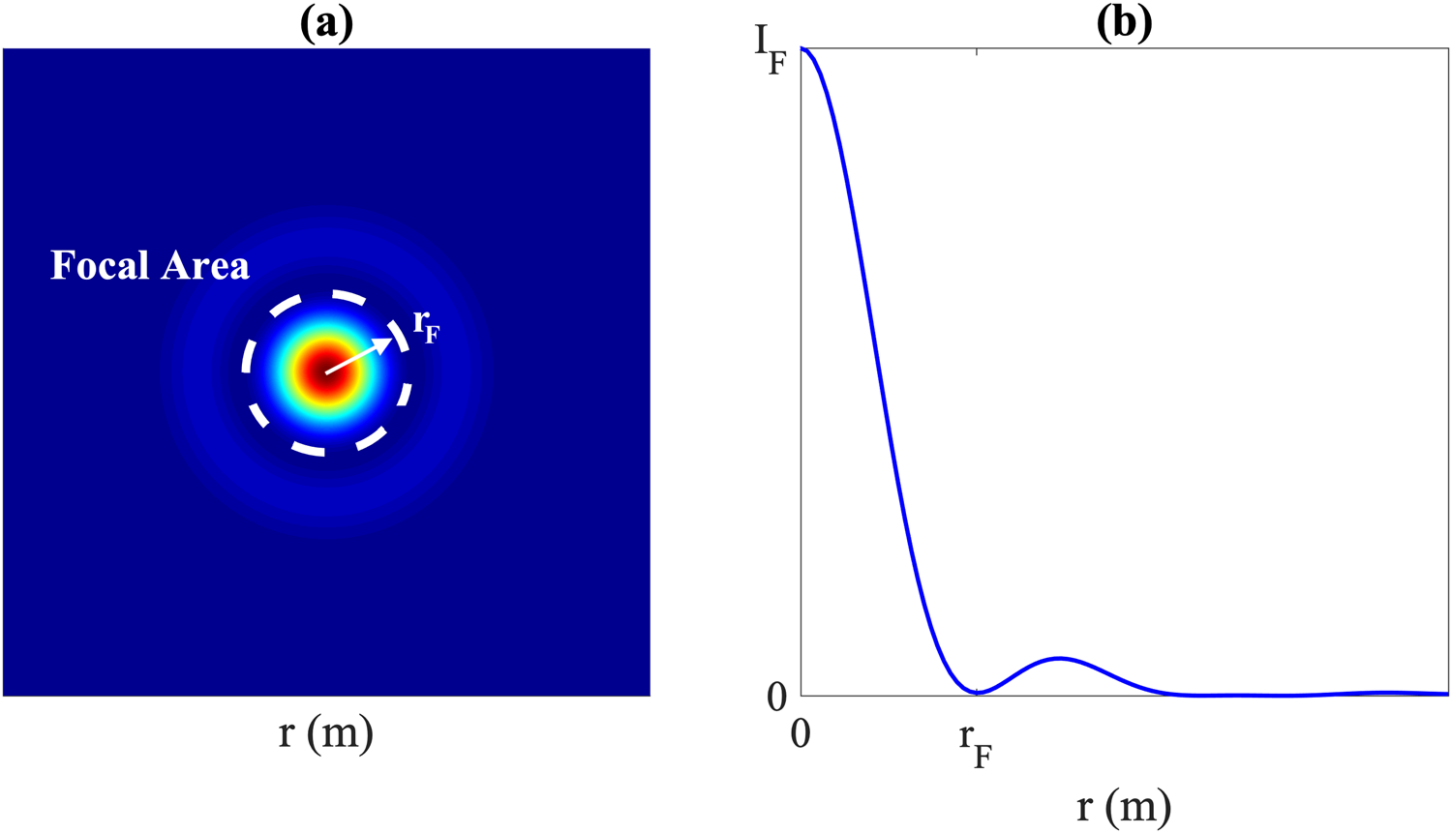
Scheme of the focusing efficiency calculation: (**a**) intensity at the focal plane and (**b**) radial intensity cut. The white dashed line marks the limit of the focal area.

As stated in the **Results** section, the focusing efficiency can be defined as the ratio between the energy at the focal area, *W*_*F*_, and the incident energy at the lens, *W*_0_. In this case, the focal area is defined as the region of the focal plane which contains the main focusing lobe, as depicted in Fig. 6. Thus, the focusing efficiency can be calculated as

5 $${\eta }_{F}=\frac{{W}_{F}}{{W}_{0}}=\frac{{\int }_{0}^{2\pi }\,{\int }_{0}^{{r}_{F}}\,I(z=F,\,r)rdrd\phi }{{\int }_{0}^{2\pi }\,{\int }_{0}^{{r}_{N}}\,{I}_{0}(z=0,\,r)rdrd\phi }=\frac{{\int }_{0}^{{r}_{F}}\,I(z=F,\,r)rdr}{{\int }_{0}^{{r}_{N}}\,{I}_{0}(z=0,\,r)rdr},$$

where *I*(*z* = *F*, *r*) represents the focal intensity, *I*_0_(*z* = 0, *r*) represents the incident intensity, *r*_*N*_ is the outer radius of the lens and *r*_*F*_ is the focal radius.

When plane wave incidence is considered, the incident pressure can be expressed as *p*_*i*_ = *p*_0_*e*^*jkz*^ and therefore the incident intensity is given by

6 $${I}_{0}(z=0,r)=\frac{|{p}_{0}{|}^{2}}{2{Z}_{0}},$$

being *p*_0_ the amplitude of the incident wave. With Eq. 6, the incident energy can be directly calculated as

7 $${W}_{0}={\int }_{0}^{2\pi }\,{\int }_{0}^{{r}_{N}}\,{I}_{0}(z=0,r)rdrd\phi =\frac{|{p}_{0}{|}^{2}\pi {r}_{N}^{2}}{2{Z}_{0}}.$$

In contrast, when a directional piston transducer placed at a distance *d* from the lens is used as emitter, the incident pressure can be expressed as

8 $${p}_{i}(r)=\frac{jk{p}_{0}{a}^{2}}{2\sqrt{{r}^{2}+{d}^{2}}}D(r){e}^{-jk\sqrt{{r}^{2}+{d}^{2}}},$$

where *p*_0_ is the pressure at the surface of the piston, *a* is the piston active radius and *D*(*r*) is the piston directivity pattern, which is given by

9 $$D(r)=\frac{2{J}_{1}(kar/\sqrt{{r}^{2}+{d}^{2}})}{kar/\sqrt{{r}^{2}+{d}^{2}}},$$

being *J*_1_ the first kind and first order Bessel function.

Therefore, for the piston transducer case the incident energy can be calculated using the following expression:

10 $${W}_{0}=\frac{|{p}_{0}{|}^{2}\pi {a}^{2}}{{Z}_{0}}{\int }_{0}^{{r}_{N}}\,\frac{1}{r}{J}_{1}{(ka\frac{r}{\sqrt{{r}^{2}+{d}^{2}}})}^{2}dr.$$

The focal intensity generated by the lens can be calculated by numerically computing Eq. 4. Once the focal intensity is obtained, the energy at the focus, *W*_*F*_, can be computed by integrating the result over the focal area. Subsequently, the focusing efficiency can be calculated using Eqs 7 and 10 for the plane wave and piston transducer case, respectively. Table 3 shows a comparison between the focusing efficiency values of an ideal FZP and an ideal PR-FZP with *N* = 16 regions and *F* = 50 mm. The ideal FZP implements perfect pressure blocking regions with |Γ| = 1, while the ideal PR-FZP implements perfect phase-reversal regions with |Γ| = 0. Both lenses have a transparent central region and a working frequency of 1 MHz. For the piston transducer case, a separation of *d* = 340 mm between lens and emitter and an active radius of *a* = 6.35 mm have been considered. As it can be observed from the results shown in Table 3, the PR-FZP achieves higher focusing efficiency than the conventional FZP in both the plane wave incidence case and the piston transducer case. Moreover, it is worth noting that in the piston transducer case both FZPs reach higher efficiency values compared to the plane wave case. This fact can be explained by the directional behaviour of the piston transducer, which concentrates the main energy contributions on the central regions of the lens.

**Table 3:** Focusing efficiency (*η*_*F*_) comparison between plane wave and piston transducer case.

	Plane wave (%)	Piston transducer (%)
FZP	9.9	12.2
PR-FZP	37.0	42.1

The experimental value of the focusing efficiency can be obtained by carrying out two different measurement steps. The first step is performed with the lens, and it consists of the measurement of the radial intensity at the focal plane, that is, *I*(*z* = *F*, *r*). The second step is performed without the lens, and it consists of the measurement of the incident intensity at the lens, *I*_0_(*z* = 0, *r*). In this measurement, the radial intensity is measured at the position where the lens was placed at the first step. Finally, the focusing efficiency can be calculated by numerically computing Eq. 5.

### Fem model explanation

**Figure 7:**
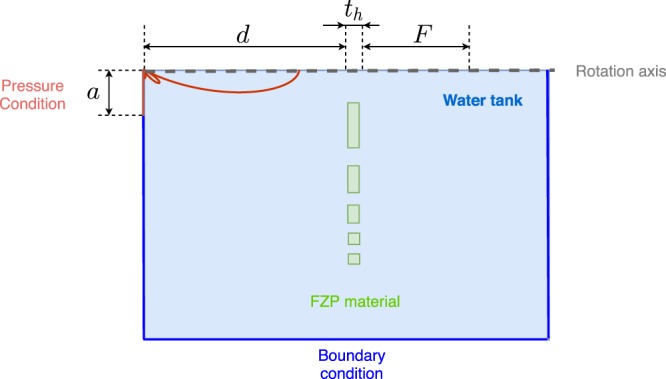
Scheme of the geometry and boundary conditions of the Finite Element Method model.

The numerical model is shown in Fig. 7 and it can be observed that only a half-plane of the lens has been considered to take advantage of the axisymmetric condition. This approach reduces the number of degrees of freedom and therefore the computational burden of the problem. The piston transducer has been modeled as a pressure condition of 1 Pa placed at a distance *d* = 340 mm from the lens. In order to avoid reflections, a radiation condition has been set at the outer boundaries in order to emulate the Sommerfeld condition. The acoustic properties of the different materials considered can be found in Table 1 of the Results section.

The study of the physical phenomena involved in the interaction between the lenses and the wavefront requires a mathematical model that considers the boundary conditions of the problem. In the present work, the commercial software COMSOL Multiphysics^32^, which implements the FEM method, has been used to calculate the acoustic pressure distribution generated by the lens. This method obtains a numerical solution by discretizing the model depicted in Fig. 7 and solving the Helmholtz Partial Differential Equation:

11 $$\nabla \cdot (-\frac{1}{\rho }(\nabla p))=\frac{{k}^{2}p}{\rho }.$$

### Experimental set-up description

**Figure 8:**
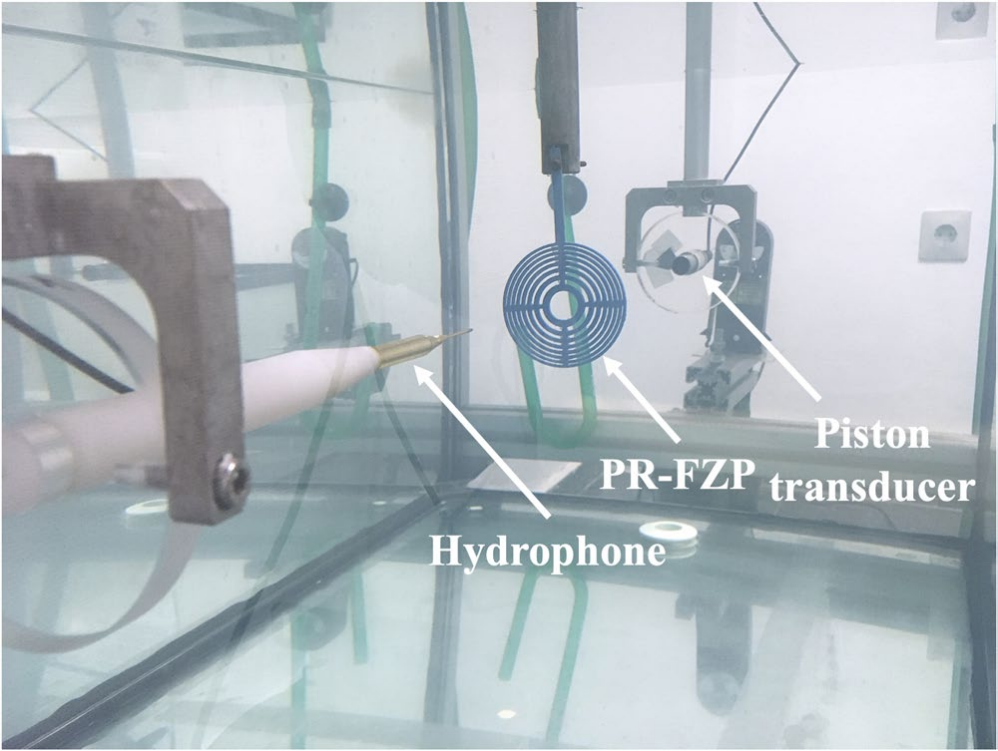
Experimental set-up.

In order to validate numerical results, experimental measures have been carried out. To perform the experimental measurements, a high-precision underwater measurement system of the Centro de Tecnologías Físicas (Universitat Politècnica de València) has been used. The measurement robot is installed in an immersion tank filled of distilled water, which dimensions are 0.5 × 0.5 × 1 m^3^. This system consists of a fixed emitter and a receiver coupled to programmable robotic arms, which can measure with a spatial resolution of 1 × 1 × 1 mm^3^. A plane immersion piston transducer built by Olympus with 1 MHz of central working frequency and an active diameter of 12.7 mm has been used as emitter and a Precision Acoustics needle hydrophone is used as receiver. The transmitted signal is generated using a Panametrics Pulser, while the received signal is digitized using a Digital Oscilloscope from Pico Technology. The measurement process is automated and controlled using a LabView program installed on a PC. Figure 8 shows the experimental set-up in a measurement.

## References

[1] Sharma, S. K., Chen, D. & Mudhoo, A. *Handbook on applications of ultrasound: sonochemistry for sustainability* (CRC press, 2011).

[2] Minin, I. V. & Minin, O. V. *Ultrasound Imaging - Medical Applications* (InTechOpen, 2011).

[3] Cervera F (2001). Refractive acoustic devices for airborne sound. Phys. Rev. Lett..

[4] Peng P, Xiao B, Wu Y (2014). Flat acoustic lens by acoustic grating with curled slits. Phys. Lett. A.

[5] Wang W, Xie Y, Konneker A, Popa B-I, Cummer SA (2014). Design and demonstration of broadband thin planar diffractive acoustic lenses. Appl. Phys. Lett..

[6] Yang X, Yin J, Yu G, Peng L, Wang N (2015). Acoustic superlens using Helmholtz-resonator-based metamaterials. Appl. Phys. Lett..

[7] Lin Z (2014). Acoustic focusing of sub-wavelength scale achieved by multiple Fabry-Perot resonance effect. J. Appl. Phys..

[8] Xia, X. *et al*. Planar ultrasonic lenses formed by concentric circular sandwiched-ring arrays. *Adv. Mater. Technol*. 1800542 (2018).

[9] Soret J (1875). Ueber die durch kreisgitter erzeugten diffractionsphänomene. Ann. Phys..

[10] Park, J. J. *et al*. Table-top soft x-ray microscope adopting a pmma phase-reversal zone plate. *In Conference on Lasers and Electro-Optics*, JFA6 (Optical Society of America, 2009).

[11] Schindel D, Bashford A, Hutchins D (1997). Focussing of ultrasonic waves in air using a micromachined Fresnel zone-plate. Ultrasonics.

[12] Welter JT (2011). Focusing of longitudinal ultrasonic waves in air with an aperiodic flat lens. J. Acoust. Soc. Am..

[13] Welter JT (2012). Broadband aperiodic air coupled ultrasonic lens. Appl. Phys. Lett..

[14] Molerón M, Serra-Garcia M, Daraio C (2014). Acoustic Fresnel lenses with extraordinary transmission. Appl. Phys. Lett..

[15] Li Y (2014). Three-dimensional ultrathin planar lenses by acoustic metamaterials. Sci. Rep..

[16] Calvo DC, Thangawng AL, Nicholas M, Layman CN (2015). Thin Fresnel zone plate lenses for focusing underwater sound. Appl. Phys. Lett..

[17] Castiñeira-Ibáñez S, Tarrazó-Serrano D, Minin OV, Rubio C, Minin IV (2019). Tunable depth of focus of acoustical pupil masked Soret zone plate. Sens. Actuators A: Phys.

[18] Tarrazó-Serrano D, Rubio C, Minin OV, Candelas P, Minin IV (2019). Manipulation of focal patterns in acoustic Soret type zone plate lens by using reference radius/phase effect. Ultrasonics.

[19] Marsac L (2012). MR-guided adaptive focusing of therapeutic ultrasound beams in the human head. Med. Phys..

[20] Herrmann K-H, Gärtner C, Güllmar D, Krämer M, Reichenbach JR (2014). 3D printing of MRI compatible components: Why every MRI research group should have a low-budget 3D printer. Med. Eng. Phys..

[21] Drumright RE, Gruber PR, Henton DE (2000). Polylactic acid technology. Adv. Mater..

[22] Ebbini ES, Cain CA (1991). A spherical-section ultrasound phased array applicator for deep localized hyperthermia. IEEE Trans. Biomed. Eng..

[23] Uchida T (2006). Treatment of localized prostate cancer using High-Intensity Focused Ultrasound. BJU International.

[24] Fuster J, Candelas P, Castiñeira-Ibáñez S, Pérez-López S, Rubio C (2017). Analysis of Fresnel zone plates focusing dependence on operating frequency. Sensors.

[25] Rayleigh, L. *Wave Theory*, vol. **24** (Encyclopedia Britannica, 1888).

[26] Black DN, Wiltse JC (1987). Millimeter-wave characteristics of phase-correcting Fresnel zone plates. IEEE Trans. Microw. Theory Tech..

[27] Huder B, Menzel W (1988). Flat printed reflector antenna for mm-wave applications. Electron. Lett..

[28] Machado F, Zagrajek P, Monsoriu JA, Furlan WD (2018). Terahertz sieves. IEEE Trans. Terahertz Sci. Technol..

[29] Kundu, T., Placko, D., Rahani, E. K., Yanagita, T. & Dao, C. M. Ultrasonic field modeling: A comparison of analytical, semi-analytical, and numerical techniques. *IEEE Trans. Ultrason., Ferroelec., Freq. Control* **57** (2010). 10.1109/TUFFC.2010.1753 21156375

[30] Pérez-López S, Fuster JM, Candelas P, Rubio C, Belmar F (2018). On the use of phase correction rings on Fresnel zone plates with ultrasound piston emitters. Appl. Phys. Lett..

[31] Takeuchi, A., Uesugi, K. & Suzuki, Y. Improvement of quantitative performance of imaging x-ray microscope by reduction of edge-enhancement effect. *J. Phys.: Conference Series*, vol. **849**, 012055 (IOP Publishing, 2017).

[32] COMSOL-Multiphysics. Comsol-multiphysics user guide (version 4.3a). *COMSOL User Guid. (version 4.3a)* 39–40 (2012).

